# Flagellar Hooks and Hook Protein FlgE Participate in Host Microbe Interactions at Immunological Level

**DOI:** 10.1038/s41598-017-01619-1

**Published:** 2017-05-03

**Authors:** Ying Shen, Lin Chen, Meixiang Wang, Dandan Lin, Zhongjie Liang, Peiqing Song, Qing Yuan, Hua Tang, Weihua Li, Kangmin Duan, Baiyan Liu, Ge Zhao, Yiqiang Wang

**Affiliations:** 1 MOH Key Lab of Thrombosis and Hemostasis, Collaborative Innovation Center of Hematology-Thrombosis and Hemostasis Group, Jiangsu Institute of Hematology, The First Affiliated Hospital of Soochow University, Suzhou, 215007 China; 2 Department of Oral Biology & Medical Microbiology, Faculty of Health Sciences, University of Manitoba, Winnipeg, Manitoba R3E 0W2 Canada; 3 Molecular Microbiology Laboratory, Northwest University, Xi’an, 710069 China; 4 Institute of Immunology, Taishan Medical University, Taian, 271000 China; 5 Shandong Provincial Key Laboratory of Ophthalmology, Shandong Eye Institute, Qingdao, 266071 China; 6 Center for Systems Biology, Soochow University, Suzhou, 215006 China; 7 Institute of Basic Medical Sciences, National Center of Biomedical Analysis, Beijing, 100850 China; 8 Key Laboratory of Internal Medicine, Hunan University of Traditional Chinese Medicine, Changsha, 410007 China; 9 China Animal Health and Epidemiology Center, Qingdao, 266114 China

## Abstract

Host-microbe interactions determine the outcome of host responses to commensal and pathogenic microbes. Previously, two epithelial cell-binding peptides were found to be homologues of two sites (B, aa168–174; F, aa303–309) in the flagellar hook protein FlgE of *Pseudomonas aeruginosa*. Tertiary modeling predicted these sites at the interface of neighboring FlgE monomers in the fully formed hook. Recombinant FlgE protein stimulated proinflammatory cytokine production in a human cell line and in murine lung organoid culture as detected with real-time RT-PCR and ELISA assays. When administered to mice, FlgE induced lung inflammation and enhanced the Th2-biased humoral response to ovalbumin. A pull-down assay performed with FlgE-saturated resin identified caveolin-1 as an FlgE-binding protein, and caveolin-1 deficiency impaired FlgE-induced inflammation and downstream Erk1/2 pathway activation in lung organoids. Intact flagellar hooks from bacteria were also proinflammatory. Mutations to sites B and F impaired bacteria motility and proinflammatory potency of FlgE without altering adjuvanticity of FlgE. These findings suggest that the flagellar hook and FlgE are novel players in host-bacterial interactions at immunological level. Further studies along this direction would provide new opportunities for understanding and management of diseases related with bacterial infection.

## Introduction

Some species of bacteria require flagellum for motility and colonization. Recent research has revealed that the flagellum can also participate in the adhesion of bacteria to host tissues^1, 2^, cells^3^ or medical device surfaces^4^. In most bacterial species, an intact flagellum, from the basal body complex to the tip, contains as many as 40 distinct types of protein^5^. The major extracellular part, namely the filament, is formed by polymerization of a protein named flagellin encoded by genes bearing different names in various bacterial species, i.e., *fliC* in *Pseudomonas* (*P*.) *aeruginosa* and *Salmonella enterica serovar* (*S. enterica*) Typhimurium, or *flaA* in *Vibrio cholerae*. Extensive studies have revealed several receptors that respond to bacterial flagellum-related stimuli. Among these, TLR5 recognizes flagellins present in the extracellular space^6, 7^, while NLRC4 and NLRP3 inflammasomes sense cytosolic flagellins derived from intracellular bacteria^8, 9^. On the contrary to flagellin, those less-abundant components of flagellum have been largely overlooked in the context of immunity or host-pathogen interactions^2^. Among these are flagellar hooks that transfer the rotation of basal body motor into propelling power of flagellum. In *P. aeruginosa*, the flagellar hook is formed by polymerization of approximately 120 single molecules named flagellar hook protein E (FlgE) (NP_249771), encoded by the flagellar hook protein gene, *flgE* (GeneID: 878285). In addition to providing flexibility and power conversion between the basal body and the filament, FlgE is also involved in the elongation or assembly of the filament, and *flgE* mutations often result in structural abnormality or deficiency in multiple bacterial species^10–13^. However, additional roles that this protein may play, as either an isolated monomer or a polymer in the hook, remain unclear except for a few reports on the antigenicity of FlgE in bacterial infections^14–16^. Thus, any clues pointing to novel biological properties of FlgE deserve attention and help to dissect host-pathogen interactions. Recently we screened a commercial 12-amino acid phage-display library (New England Biolabs, Beveraly, MA) against an SV40-immortalized human corneal epithelial cell line (HCECs) to search for potential ligands that bind epithelial cells^17, 18^. We identified two peptides, VATPVPPTLTPF (Pc-B) and TPPTYSWFTHRM (Pc-F), that were highly homologous to *P. aeruginosa* PAO1 FlgE at aa168–174 (PPTVTPF, hereafter named “site B”) and aa303–309 (TPPTYAW, “site F”), respectively (Fig. S1). For both peptides, the coverages of homology were 58% (i.e. 7aa/12aa) and identities 87% (i.e. 6aa/7aa). We interpreted these findings to indicate FlgE may interact with certain molecule(s) on the surfaces of epithelial cells. Since *P. aeruginosa* infections are common endangers to both ocular surface and respiratory system^19^, we anticipated that pursuit along this hypothesis might shed light on pathogenesis of disorders like bacterial keratitis or pneumonia. A pilot study showed that recombinant PAO1 hook protein FlgE stimulated HCECs to secrete IL6 and IL8^20^, strongly suggesting a proinflammatory effect of FlgE proteins. In the current study, we use wild-type (WT) and mutated recombinant FlgE proteins as well as purified flagellar hooks of PAO1 strains to show that both FlgE monomers and naturally existing polymers are potent proinflammatory or immunostimulatory agents, revealing FlgE and the flagellar hook as novel mediators of host-microbe interactions.

## Results

### FlgE Activates Proinflammatory Pathways in HCECs

The corneal epithelium is a site susceptible to microbial infections, and in some areas^21^ or in certain seasons^22^ *P. aeruginosa* is the leading cause of bacterial keratitis. Thus, HCECs were selected for studying the hypothetical bioactivity of PAO1 FlgE *in vitro*. A recombinant protein FlgE corresponding to the PAO1 *flgE* gene product (NP_249771) was expressed using pET24a vector in *Escherichia coli* and purified via its 6 × His-tag at C-terminal. Prompted by the preliminary finding that FlgE stimulated IL6 and IL8 expression in HCECs^20^, we selected a human genomic expression microarray platform to profile the changes in genes or pathways in HCECs after a 4 h treatment with 20 μg/mL FlgE. The data were deposited in the NCBI Gene Expression Omnibus with an accession number GSE58422. Among all 42,545 features printed on the microarray, 22,242 protein-encoding transcripts were determined to be “detected” in at least two of the three duplicating arrays. Of the detected transcripts, 304 were up-regulated and 252 were down-regulated, defined as an FlgE treatment to control ratio of greater than 1.50-fold or less than 0.667, respectively, with a P < 0.10 for measured intensity values in these two groups. Annotation and clustering was performed by using the Database for Annotation, Visualization and Integrated Discovery (DAVID) program (NIAID, NIH), which showed that six Kyoto Encyclopedia of Genes and Genomes (KEGG) pathways were significantly (P < 0.01) enriched among the up-regulated genes (Table S1). Among these pathways, most of them were closely related to inflammation or immunity, suggesting that FlgE stimulation initiated an inflammation/immune response in HCECs. In contrast to the multiple pathways enriched among the up-regulated genes, not any pathway was significantly enriched in all 252 down-regulated probes (Table S1). It was also interesting to note that 27 of the 304 up-regulated features (8.9%) were long non-coding RNAs (LncRNAs), while 27.4% (i.e., 69 of 252) of the down-regulated features were LncRNAs (refer to GSE58422). Those genes with greater than 2-fold changes are listed in Table S2 for further reference. Among these, *CXCL1*, *IL1β* and *IL6* showed highest fold changes, i.e. 54.66 ± 6.79, 8.78 ± 0.97 and 8.28 ± 1.82 (n = 3), respectively. Since they were also typical inflammatory factors, they were chosen as representatives of such factors in most of the subsequent functional studies.

### The Stimulatory Activity of FlgE Is Abrogated by Mutations

**Figure 1:**
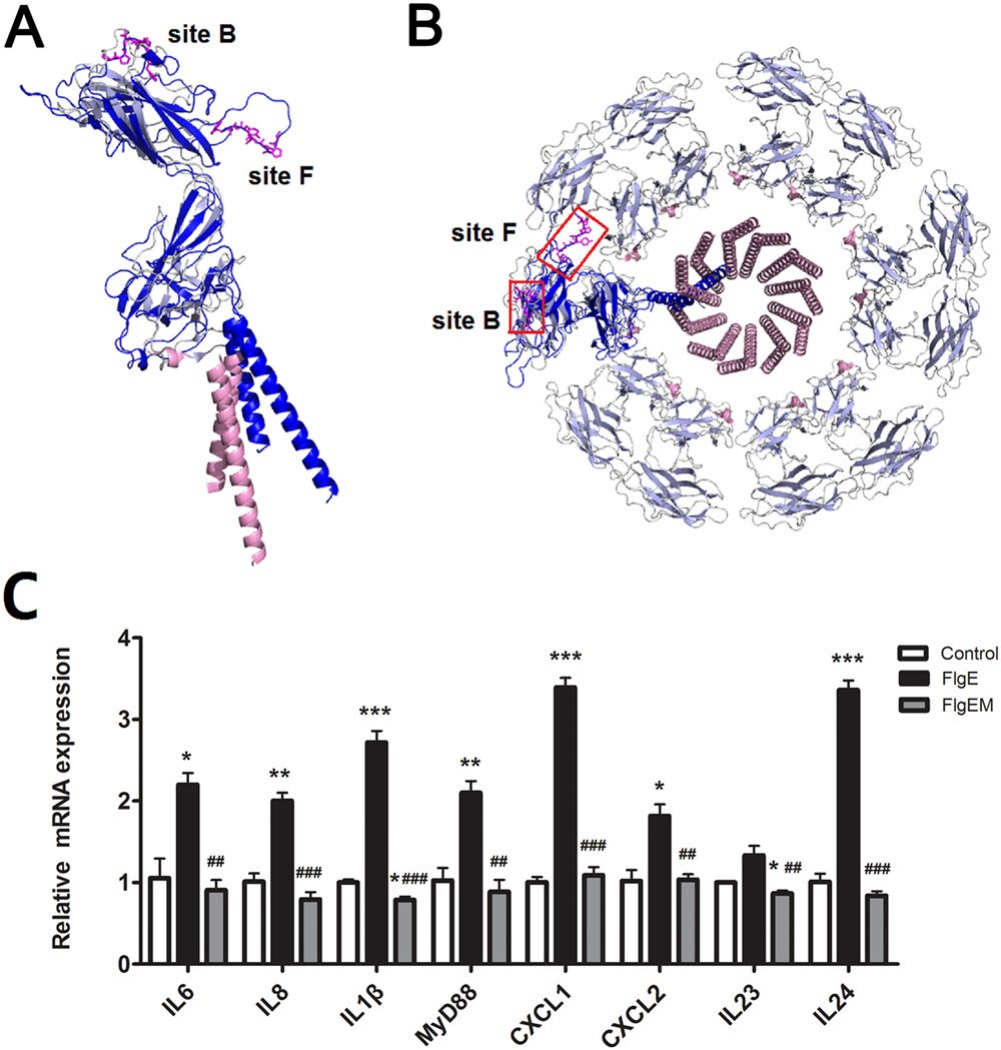
FlgE in Flagellar Hook Construction **(A,B)** and Structural Requirement for Proinflammatory Potency (**C**). (**A**) Structural alignment of the PAO1 FlgE monomer (ribbon or thread colored in blue) with FlgE from *S. typhimurium* (PDB ID code: 3A69, pink/gray ribbon/thread). The residues at the B and F sites are shown as pink sticks. (**B**) Structural setting of the PAO1 FlgE monomer (blue) over a layer of FlgE in the flagellar hook of *S. typhimurium* (pink/grey). The B and F sites are highlighted in red boxes. (**C**) Requirement of the B and F sites for FlgE induced inflammation in epithelial cells. To generate FlgEM, site B was deleted, and site F was substituted with AAA. Recombinant FlgE and FlgEM were added to HCECs at 20 μg/mL and were cultured for 4 h. RT-qPCR was performed using TaqMan probes, and the relative expression level of each gene in each sample was normalized against the reference gene and then calculated as 2^−ΔΔCt^, where ΔΔCt = ΔCt_FlgE_ − ΔCt_Control_. *P < 0.05, **P < 0.01, ***P < 0.001 vs. Control. ^#^P < 0.05, ^##^P < 0.01, ^###^P < 0.001 vs. FlgE.

Because the assumed binding of FlgE to epithelial cells was first suggested by the homology of two phage-displayed peptides to FlgE at sites B (aa168–174) and F (aa303–309), we assessed whether these two sites have any significance in the structural basis of FlgE activity. Tertiary structure modeling to *S. enterica* serovar Typhimurium FlgE of PAO1 FlgE predicted that both sites were located on the surface of a single FlgE molecule (Fig. 1A,B), validating the accessibility to FlgE at these two sites by other molecules. To determine whether these two sites are important for FlgE stimulation, a recombinant mutant FlgE (FlgEM) was designed based on structural analysis and produced in aforementioned recombinant expression system. Specifically, the 7 amino acids corresponding to site B (PPTVTPF) were deleted, and the 7 amino acid sequence of site F (TPPTYAW) was substituted with an alanine string (AAA). This designing strategy for FlgEM was chosen to minimize the possibility of altering the overall 3D structure of the whole molecule, and at the same time to maximize the chance of altering the function of FlgE, if these two sites were actually critical for FlgE function at all. Tertiary structure modeling did not reveal significant alterations in the structure of the FlgEM monomer compared to the FlgE monomer (data not shown). However, when added to HCEC culture, unlike FlgE, FlgEM did not alter the expression of any of the eight inflammation-related genes (Fig. 1C). The deleterious effect of the two mutations in FlgEM suggests that sites B and F may be critical for interactions between FlgE and cellular components in *in vitro* proinflammation setting.

### FlgE Elicits a Proinflammatory Response in Mice

**Figure 2:**
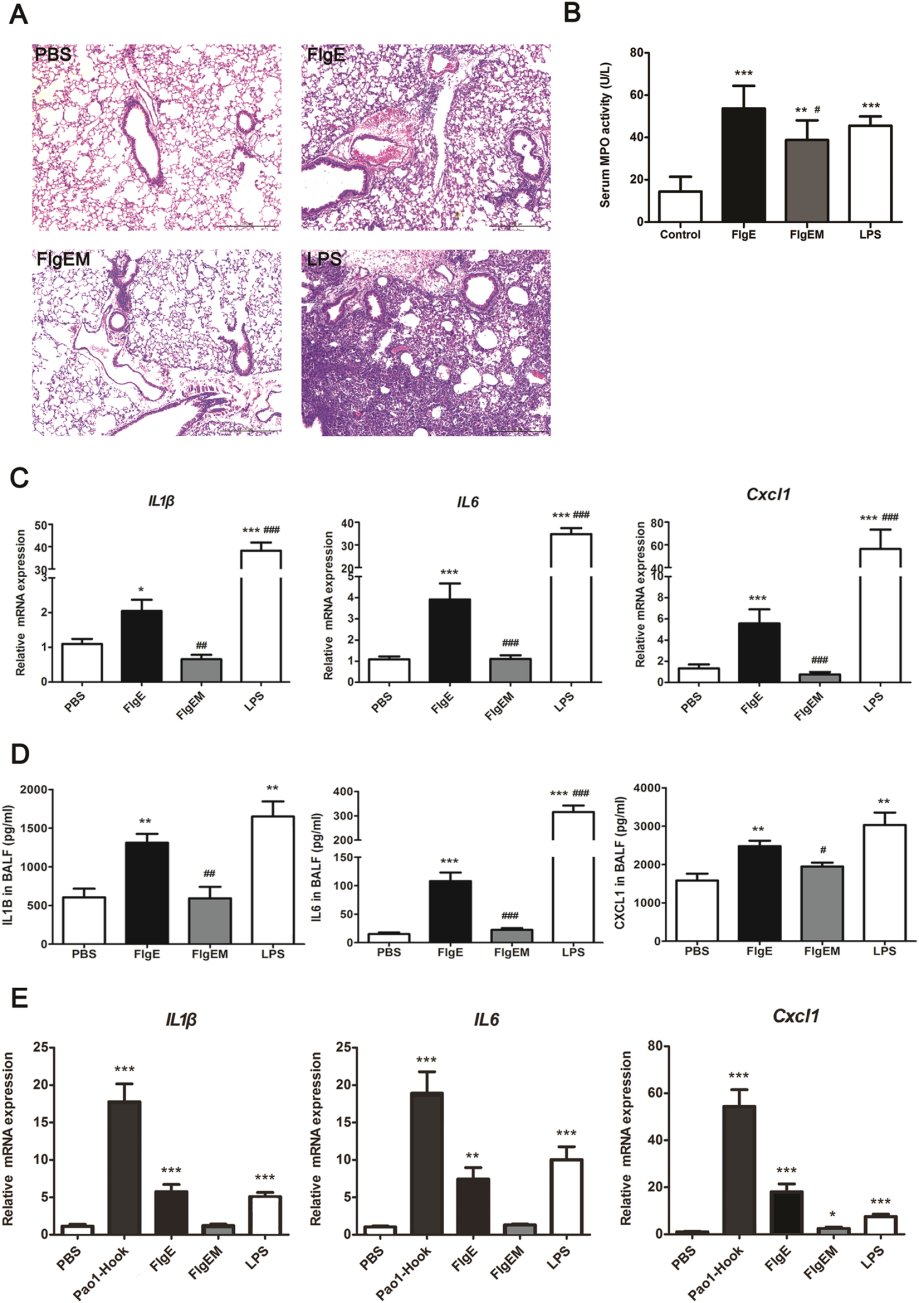
Proinflammatory Activity of FlgE in Mice **(A–D)** or Lung Organoid (**E**). (**A**–**D**) C57BL/6 mice were intranasally administered 120 μg recombinant FlgE, FlgEM, or LPS in a 150-μL volume. Twenty-four hours later, the animals were sacrificed by bleeding under anesthesia, and bronchoalveolar lavage was performed before the lungs were cut into halves that were subjected to either fixation for hematoxylin/eosin staining (**A**) or to RNA extraction for RT-qPCR (**C**). Quantification of chemokines in the lavage was by ELISA (**D**), and myeloperoxidase (MPO) levels in the serum were measured using an MPO determination kit (**B**). Data are representative of two independent experiments that showed similar results. N = 6 mice for each treatment group. (**E**) For lung organoid stimulation, lung slices were treated with various reagents for 24 hours and then subjected for chemokine mRNA measurement with RT-qPCR. Data are representative of three independent experiments. Three mice were used for making mixture of lung organoid cultures. Two samples were analyzed for each group. *P < 0.05, **P < 0.01, ***P < 0.001 vs. Control/PBS. ^#^P < 0.05, ^##^P < 0.01, ^###^P < 0.001 vs. FlgE.

Next, we measured the proinflammatory potency of FlgE *in vivo* using murine models. Intranasal administration of FlgE to mice induced an inflammatory response in the lungs, indicated by universal infiltration of leukocytes (Fig. 2A) and an increase in the myeloperoxidase (MPO) level in serum (Fig. 2B). Supporting these observations, FlgE treatment resulted in increases in IL1β, IL6 and CXCL1 at the mRNA level in lung tissues (Fig. 2C) and at the protein level in bronchoalveolar lavage fluid (Fig. 2D). In contrast, when FlgEM was used in this model, the inflammatory infiltration and the cytokine production observed for FlgE were absent (Fig. 2A,C,D), but the MPO elevation in serum retained to certain extent (Fig. 2B), indicating a segregation of pathways for stimulating chemokine production and for neutrophil activation. As another control, ethanol-denatured form of FlgE was entirely devoid of proinflammatory activity (Fig. S2). All these data showed that native FlgE was highly proinflammatory and alteration of structure in any forms might abrogate this property. More striking was that the polymerized format of FlgE, i.e. the flagellar hook from natural bacteria, was more efficient than same mass concentration of FlgE monomers in a stimulating inflammation in lung organoid culture model (Fig. 2E).

### FlgE Elicits an Adjuvant Potential in Mice

**Figure 3:**
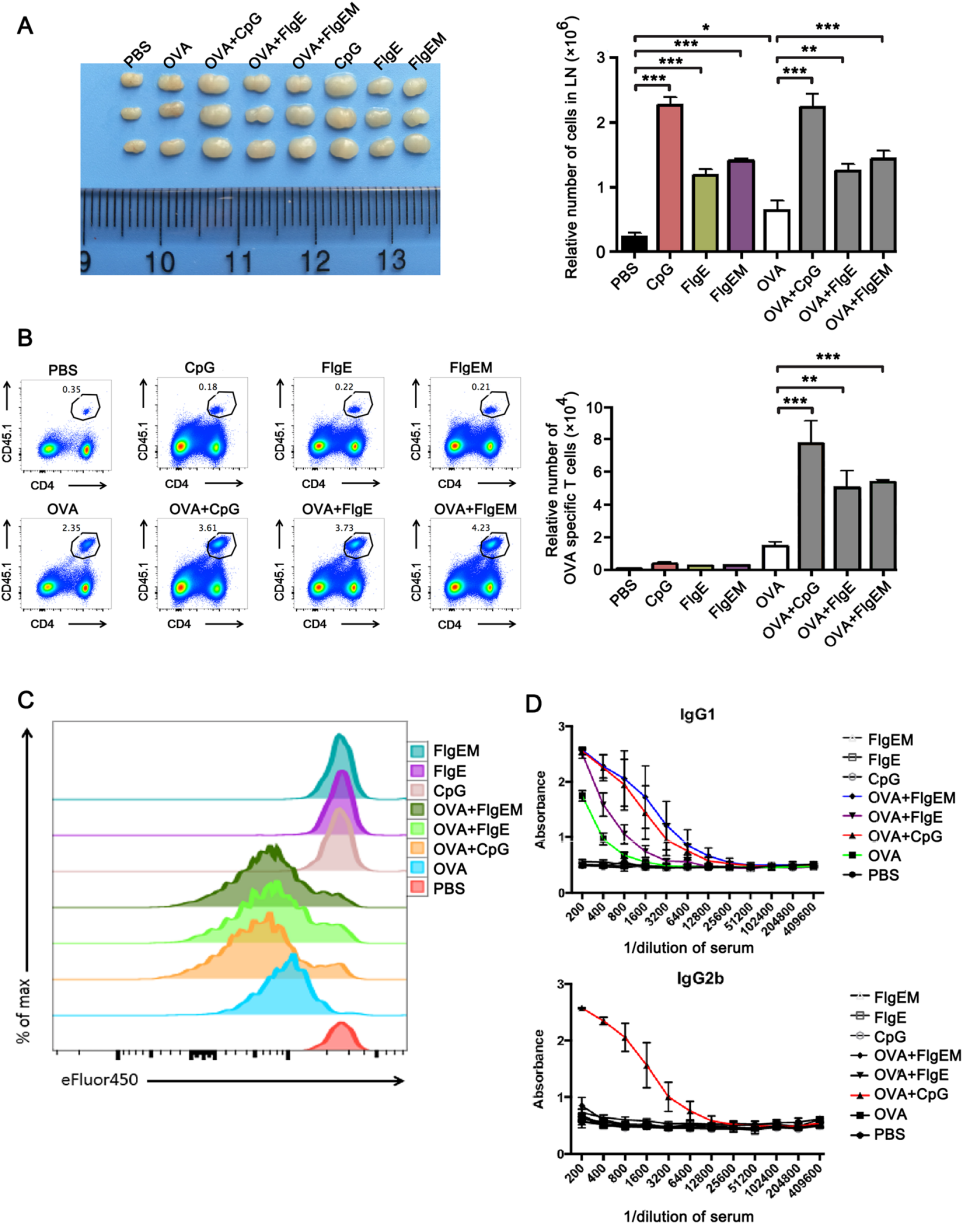
Adjuvanticity of FlgE to Soluble Antigen in Mice. (**A–C**) Naïve OVA-specific T cells were purified from the spleens and inguinal lymph nodes of CD45.1 × OTII F1 mice by sorting using various antibodies to surface markers, labeled with eFluor450 and transferred into female C57BL/6 mice at 1 × 10^6^ cells/mouse via tail vein injection. Twenty-four hours later, the recipients were treated or immunized via subcutaneous injection at the base of the tail with one of eight compositions, namely 1 μg OVA, 50 μg CpG-1826, 50 μg FlgE, 50 μg FlgEM each alone, or OVA plus the three stimulants individually. Control groups received 100 μL PBS. Three days later, the mice were sacrificed, and the draining inguinal lymph nodes were isolated and photographed (**A**). Single-cell suspension were made for staining with CD45.1 plus CD4 and then run on a BD FACSAria II and analyzed for eFluor450 intensity to quantify the proliferation of OVA-specific T cells (**B,C**). (**D**) To measure the humoral response, WT C57BL/6 mice were immunized with above 8 compositions except for increasing OVA doses to 100 μg. Two weeks later, the mice were sacrificed for serum harvest, and anti-OVA titers were measured using an ELISA as described in Materials and Methods. Data are representatives of two independent experiments that showed similar results. n = 3 mice for panel A–C, and n = 4 mice for panel D. *P < 0.05, **P < 0.01, ***P < 0.001.

Since for many microbial products/components like flagellin or lipopolysaccharide (LPS) their proinflammatory potencies are accompanied with a potential to stimulate or modulate host adaptive immune responses to other antigens, we continued to check if FlgE could also be an adjuvant for immunizing antigens in mice. OVA was chosen as a soluble antigen in an OVA-specific TCR transgenic mice model, with CpG motif containing oligodeoxynucleotides (CpG) as confirmed adjuvant control. When FlgE, FlgEM or CpG were administered with OVA to mice via subcutaneous injection, they enhanced OVA-specific T lymphocyte proliferation and increased the mass of draining lymph nodes at day three (Fig. 3A–C). When these three agents were administered alone without OVA, though they also stimulated draining lymph node expansion, supposedly via proinflammation, they did not alter the number of OVA-specific T cells proliferation. It was worthy to point out that in this context, FlgEM was slightly more effective than FlgE. In another experimental setting where anti-OVA antibodies were looked at, FlgE, FlgEM and CpG all enhanced the production of IgG1 class anti-OVA antibody, but only CpG increased production of IgG2b class antibody (Fig. 3D), implying that a Th2 type-dominant immune response was induced by FlgE or FlgEM treatment, while CpG enhanced both Th1 and Th2 type immune responses just as commonly known.

### Caveolin-1 Helps Mediate FlgE Stimulation in Epithelial Cells

**Figure 4:**
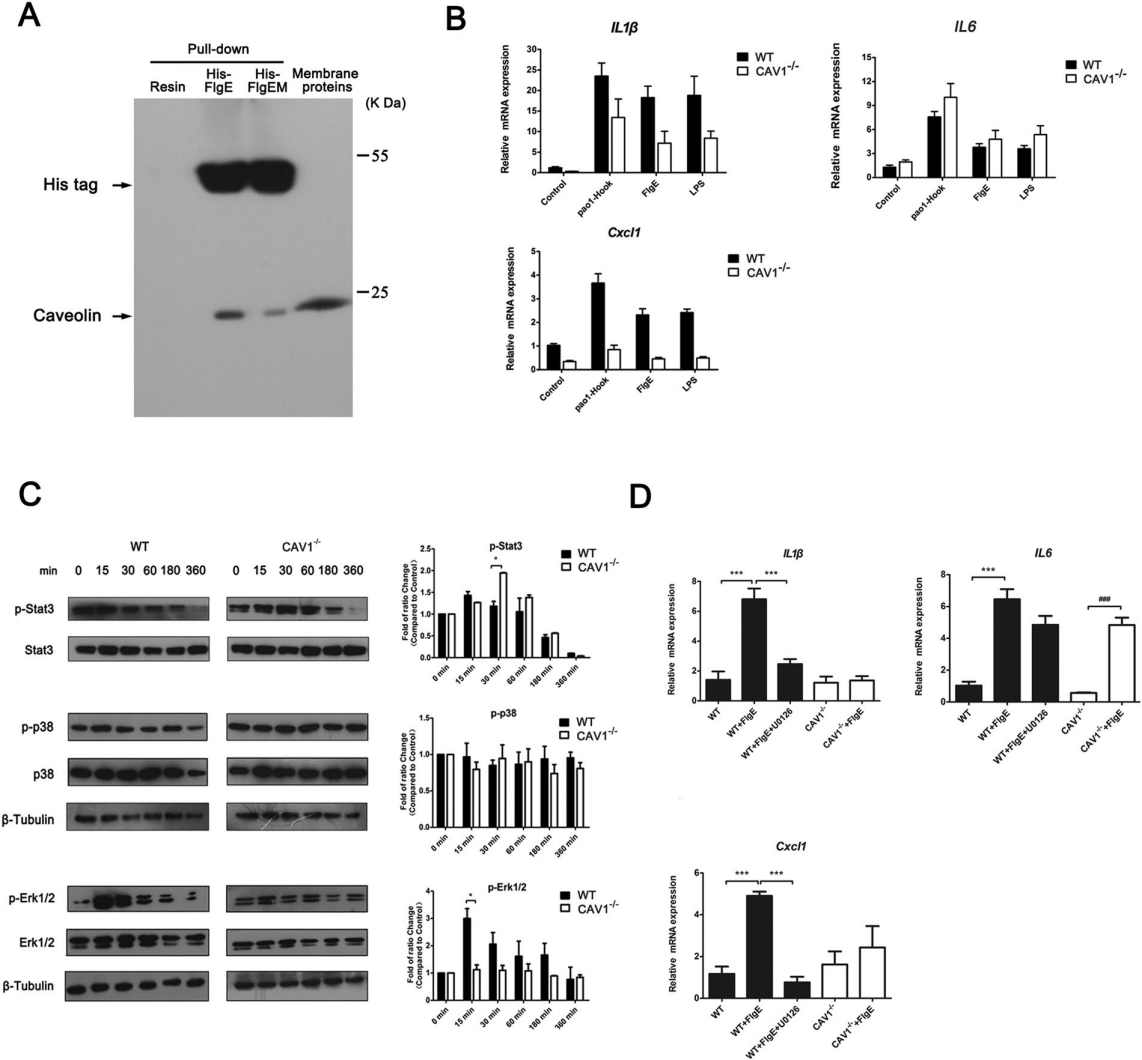
Caveolin-1 as a Potential Mediator of FlgE Stimulation via the MEK-Erk Pathway. (**A**) Confirmation of CAV1 presence among HCEC membrane proteins and among proteins pulled down by the FlgE or FlgEM-conjugated resin. The pull-down procedure was also performed with a “resin” control that was not conjugated with FlgE. (**B**) Impairment of IL1β and Cxcl1 expression induced by FlgE (5 μg/mL), the flagellar hook (5 μg/mL) or LPS (5 μg/mL) in organoid cultures of CAV1^−/−^ mice lungs compared with that in WT mouse lungs. Please note that IL6 expression induced by these agonists was not hindered by CAV1 deficiency. RT-qPCR was performed using TaqMan probes, and the mRNA expression level of each gene is relative to the expression level of that gene in WT mice. Data are representative of two independent experiments. Three mice were used for making lung slices that were evenly distributed into each treatment wells, and two duplicate wells were set for each treatment. *P < 0.05, **P < 0.01, ***P < 0.001 between WT and CAV1^−/−^. (**C**) Involvement of Erk and Stat3 pathways in FlgE stimulation in organoids that were blocked by CAV1 deficiency. For densitometry, the intensity of each gene at each time point was normalized against sample at 0 minute. Data are presented as the means ± SEM, n = 2. (**D**) A unique MEK inhibitor, U0126 (10 µM), was added to WT organoids 30 min before the addition of FlgE to further confirm the involvement of the MEK-Erk pathway in FlgE stimulation. Data are representative of three independent experiments that yielded similar results. N = 3 mice for each group of lung organoid cultures. Two samples were analyzed in each group. *P < 0.05, **P < 0.01, ***P < 0.001, between WT and CAV1^−/−^.

Lastly, efforts were made to identify possible receptors or coupling proteins involved in FlgE stimulation. Quite understandably, different cells might utilize different receptors/pathways to convey FlgE stimuli, and we focused on HCEC cells without going into other cells such as those involved in mediating FlgE adjuvanticity. An affinity chromatography strategy was employed using a 6 × His-FlgE-conjugated nickel column to screen for interacting candidates in bulk proteins prepared from HCEC lysates. Mass spectrum detection of the pulled-down fractions and subsequent Mascot analysis suggested a total of 17 proteins as potential FlgE-binding partners, some of which clustered into protein families such as ATP synthase, cytokeratin, and Ras-related proteins (Table 1). None of these 17 candidates strongly correlated with the well-known TLR-MyD88 axis that mediates recognition of most pathogen-Associated molecular patterns (PAMP), implying that TLRs might not be sole receptors for FlgE recognition. Because caveolin-1 (CAV1) mediates host responses to *Pseudomonas* ^23, 24^ and other infections, we focused on CAV1 for further confirmation studies. Western blots of the bulk HCEC lysates and FlgE pulled-down fractions showed that CAV1 was enriched in the latter samples, while FlgEM was less efficient in pulling-down CAV1 (Fig. 4A). In the lung organoid culture of tissues from CAV1-deficient (CAV1^−/−^) and WT mice, the stimulatory effect of FlgE was impaired in CAV1^−/−^ mice, as indicated by decreases in CXCL1 and IL1β mRNA levels and elevated IL6 mRNA levels (Fig. 4B). While the changes in the expression patterns of CXCL1 and IL1β in these tissues are readily explained, the opposite change in IL6 mRNA expression in same condition was seldom noted, though it is in line with a recent observation that blocking CAV1 expression in chondrocytes with CAV1-siRNA increased IL6 production^25^. The CAV1-dependent effect with FlgE-induced inflammatory cytokine production was also observed in organoids treated with intact hooks from PAO1 (Fig. 4B). Furthermore, in lung organoids from CAV1^−/−^ mice, intact hook stimulated more cytokine production than the recombinant FlgE monomers of same mass concentrations (Fig. 4B), just like in lung organoids from WT mice (Fig. 2E).

**Table 1:** Summary of proteins that were pulled-down by FlgE proteins.

GI	Gene Description	Scores and Matches*
32189394	ATP synthase subunit beta, mitochondrial precursor	1095, 32(25); 311, 15(7); 204, 9(5)
4757810	ATP synthase subunit alpha, mitochondrial isoform a precursor	733, 41(19); 145, 5(4)
4502303	ATP synthase subunit O, mitochondrial precursor	212, 10(5)
38516	Caveolin-1	290, 12(7)
10120511	Chain A, crystal structure of the human hydroxysteroid sulfotransferase in the presence of Pap	320, 9(6); 229, 8(5)
289526799	Chain A, structure of the heparin-induced E1-dimer of the amyloid preprotein (App)	127, 5(3)
181400	Cytokeratin 8	186, 15(8)
435476	Cytokeratin 9	152, 6(3); 115, 9(4)
181402	Epidermal cytokeratin 2	132, 4(3)
4505773	Prohibitin	141, 11(3)
508285	Rab5c-like protein, similar to *Canis familiaris* Rab5c protein, PIR Accession Number S38625	330, 11(8)
4506365	Ras-related protein Rab-2A isoform a	318, 12(6)
20147713	Ras family small GTP binding protein RALA	244, 8(6)
4506371	Ras-related protein Rab-5B isoform 1	190, 6(4)
19923231	Ras-related protein Rab-6A isoform a	150, 14(5)
4506405	Ras-related protein Ral-B	126, 5(3)
19923262	Ras-related protein Rab-5A	121, 5(4)

*A MASCOT score at or above 114 was considered positive in this experiment. For those proteins that were identified in two or more pull-down samples, the scores and matches for each sample are separated by a semi-colon.

To further explore the possible pathways affected by FlgE stimulation under these conditions, Western blot assays were utilized to measure the activation/phosphorylation of Stat3, Erk1/2 and p38 (Fig. 4C), representative molecules belonging to the pathways revealed by previous microarray profiling of HCECs (Table S1). In organoids from WT mice, FlgE induced a transient but significant activation of Erk1/2 at 15 min that then gradually declined over the 6 h study period. However, CAV1 deficiency abrogated the change in p-Erk1/2 levels (Fig. 4C). The activation of Stat3 upon FlgE treatment in WT lung culture was much less, but in CAV1^−/−^ organoids, FlgE induced a significant increase of p-Stat3 from 15 min to 30 min that was also followed by a decrease in starting from 1 h. No significant changes in p38 phosphorylation were observed in response to FlgE treatment in either WT or CAV1-deficient tissues. As a result, FlgE-stimulated *IL1β* and *Cxcl1* overexpression was abolished by CAV1 deficiency, but *IL6* overexpression was not affected (Fig. 4D), supporting the notion that FlgE-dependent induction of cytokines occurs via different mechanisms for these cytokines. To further confirm the involvement of the CAV1/Erk pathway in FlgE stimulation, a unique MEK inhibitor, U0126, was added together with FlgE to the WT organoids, and induction of *IL1β* and *Cxcl1*, but not of *IL6*, was significantly inhibited by U0126 (Fig. 4D). Taken together, these data implied that CAV1 was one of, but not the only, initiator of pathways that mediate FlgE-initiated stimulation.

## Discussion

While previous studies mainly addressed the significance of FlgE in the bacterial flagellar structure or the antigenicity of FlgE in bacterial infections, this study is the first to show the potency of proinflammation or immunostimulation for FlgE monomers and their polymerized form in flagellar hooks. Surely this finding brought more questions than it answered, especially with the mechanisms of FlgE stimulation at different levels, among which some are briefly discussed below. The bacterial pole bearing the flagellar hook is actively involved in bacterial attachment to hydrophobic surfaces^4^. Should this also be the case for bacterial attachment to host cells, the flagellar hooks may bind to their host membrane counterparts and elicit biological reactions including inflammation/immune responses. Pull-down assays with HCECs and functional studies in CAV1^−/−^ lung organoid cultures confirmed that CAV1 was one such counterpart mediating the FlgE response. In line with the current study, CAV1 has been reported to participate in anti-microbial immune responses by facilitating the entry of microbial particles into host cells^23, 24, 26^. Though not further confirmed like with CAV1, other molecules suggested by the pull-down assay, such as ATP synthase, Rab family members, and prohibitin (Table 1), should not be overlooked in future studies since all of them have been documented to participate in host cell-microbial interactions under certain conditions. Additionally, due to the close relationship between FlgE and the filament flagellin, it is tempting to speculate that FlgE may be one type of PAMP or microbe-associated molecular pattern and may function through a more immune-like pathway as suggested by microarray data (Table S1). Actually, recent years witness progression in dissecting interactions between TLRs and non-TLR molecules, such as caveolin-1 in stimulation of traditional PAMP like lipopolysaccharide^27^, demonstrating alternative mechanisms for PAMP biology. This said, more extensive studies are needed to integrate the results of the current functional studies, the pull-down assay data and the microarray data to solidify the identity of FlgE as a PAMP and to gain a mechanistic understanding of FlgE activity.

The loss-of-function observed for recombinant FlgEM in HCECs or nasal challenged lung tissues implied that very likely site B or site F or both are involved in direct binding of PAO1 FlgE to the epithelial cells, in which a proinflammatory response is initiated. Considering the fact that the sequences of these two sites first caught our attention because of their homologies to phage-displayed peptides bound to HCEC^17^, it is likely that these two sites are involved in direct binding to sites on epithelial cells. Furthermore, 3D modeling of PAO1 FlgE based on the alignment with the crystal structure of *S*. Typhimurium FlgE^28, 29^ demonstrated that both sites are located on the surface loop areas of the FlgE monomer (Fig. 1), conferring them the capacity to interact with host counterparts. Still, the observation that the intact hook preparation from WT PAO1 was more efficient in stimulating epithelial cells than FlgE monomers of the same mass concentrations (Figs 2B and 4B) requested cautious explanations. We proposed that flagellar hooks may own a better physiochemical stability but, once released from live bacteria or after entering host cells, quickly depolymerize, allowing sites B and F to act on their receptors. This hypothesis borrowed support from studies on FliC-TLR5 interactions. FliC segments that are recognized by TLR5 are buried in the flagellar filament as well, and the filament must first be degraded into FliC monomers for TLR5 stimulation^30–32^. While occasional studies proposed that native flagella could not initiate an immune response in a way the FliC monomers did^30, 33^, some other investigations found that intact flagellar filament did initiate this response, or even more efficiently than monomers when at higher doses/concentrations^34–38^. Furthermore, FlgE has been reported to regulate flagellar development, and mutations in this gene caused flagellar deficiency and rotating impairment^10, 12, 13, 39^. Pilot studies showed that mutations with sites B and F, when introduced into PAO1 genome via recombination, impaired assembly of flagella (Fig. S3A). Even though a portion of PAO1/*flgEΔBmF* mutants grew flagella, their swimming ability was entirely absent (Fig. S3B), implying that sites B and F not only participate in interactions between FlgE and host counterparts, but also are critical for physical and mechanistic normality of flagellar hook. In one word, future comparison of the efficacies or mechanisms of intact flagellar and FlgE monomers stimulation, as well as the function-structure relationship in biological or physical aspects, will help to dissect the contribution of flagellar hook in a natural infection situations.

Along these lines, it is worth mentioning that an apparently high variety of FlgE sequences exists among different species, just like the case of filament flagellins in various species (Fig. S1, and ref. 40). Standard BLAST analysis of NP_249771 (PAO1 FlgE) against the non-redundant protein sequences database revealed that FlgE sequences are highly variable in all proteins that bear official names containing the phrase “flagellar hook protein FlgE” (not shown). Clustal alignment of NP_249771 with FlgE molecules from *S*. Typhi and *Campylobacter jejuni* indicated identity matrixes of 41.25% and 29.65%, respectively, and neither site B nor site F was conserved in these three FlgE sequences (Fig. S1). Similarly, Luneberg previously reported that FlgE from different strains of a single *Campylobacter jejuni* species manifested hypervariability in the central surface-exposed region^41^. Thus, the applicability of the structural basis for PAO1 FlgE stimulation (e.g. sites B and F) also deserves experimental examination in other genera.

Another issue deserving a brief discussion was about the adjuvanticity of FlgE. To date, most of the biological adjuvants used or explored in various vaccines have stemmed from intact microbes or their components, including FliC, nonmethylated CpG DNA and toxins, to name a few (reviews^42–44^). The current study adds FlgE to this list based on its efficacy in enhancing an adaptive immunity to antigens (Fig. 3). However, it looked like that the adjuvanticity of FlgE was not achieved via its proinflammatory function since the non-proinflammatory FlgEM was stronger than FlgE in enhancing anti-OVA antibodies production (Fig. 3D). In a recent review, Awate *et al*. summarized the mechanisms for adjuvant action as “formation of [antigenic] depot, induction of cytokines and chemokines, recruitment of immune cells, enhancement of antigen uptake and presentation, and promoting antigen transport to draining lymph nodes”^45^. A specific adjuvant might utilize one sole mechanism or a combination of above. Without going too far from our data, we proposed that even though FlgE induced cytokines and chemokines production, the adjuvanticity of FlgE or FlgEM observed in this setting should not rely much on this mechanism. Rather, they might achieve adjuvanticity, toward a Th2-dominant humoral response for OVA in this case (Fig. 3D), via other routes that were not affected by sites B/F mutations. Despite the uncertainty of the mechanisms, the adjuvant potential makes FlgE a promising candidate for vaccination or therapy of diseases related with flagellated bacteria, such as those in transplant recipients and immune-compromised patients. A recent clinical study found that a bivalent flagella vaccine (type a0a1a2 and type b), which mainly contains FliC flagellins, induced protective immunity against *Pseudomonas* infection in cystic fibrosis patients^46^. Supported by earlier proof of the antigenicity of FlgE^14–16^, our study provided new evidence that FlgE might be utilized as a preventive or therapeutic tool for combating bacterial infection.

To summarize, the current study discovered novel functions for the FlgE monomer and its polymerized form in the flagellar hook in the context of immunity. Future studies on FlgE immunostimulation should extend the current work from epithelial cells to immune cells (i.e., dendritic cells and macrophages), from the current soluble antigen immunization models to cellular antigenic models (i.e., tumor cells), and from the current PAO1 FlgE to FlgE molecules of other bacteria. Detailed mechanisms for FlgE stimulation, including proposed receptors such as CAV1, yet-to-be-determined ATP synthase/Rabs/prohibitin, MyD88 and inflammasome pathways, also deserve more extensive investigations, as do their interactions with other pathways. Further study on FlgE would shed new light on host-microbe interactions and would provide new opportunities for disease management based on such discoveries.

## Material and Methods

### Study Design

WT and mutant FlgE proteins corresponding to the FlgE protein of *P. aeruginosa* were cloned and expressed in *E. coli* followed by affinity purification and endotoxin removal. These recombinant proteins were used for treatment of HCECs, murine lung slices, or mice. To study FlgE function in the flagellar hook, WT *P. aeruginosa* was subjected to mutation of FlgE to produce three mutants representing different mutations. The effects of the mutations on flagella formation or bacterial motilities were monitored. Hooks were purified from bacteria and their immunostimulatory effects were assessed as for the recombinant FlgE protein. The main readouts in all studies include expression at the mRNA or protein levels, as detected by microarray and RT-qPCR or ELISA, respectively. To identify possible receptors or coupling molecules for FlgE stimulation, affinity chromatography was used to pull down FlgE interacting proteins, which were subsequently subjected to mass-spectral analysis. The most promising candidate, here CAV1, was further confirmed in CAV1-deficient mice. Three-dimensional modeling of FlgE and FlgEM was performed to understand the structural basis of FlgE function.

### Animals

Animal experimental protocols were approved by the Ethics Committee of Soochow University and followed the Guidelines on the Humane Treatment of Laboratory Animals (Ministry of Science and Technology of China, 2006). C57BL/6 mice were purchased from Vital River Laboratory Animal Technology Co. (Beijing, China). CAV1-deficient (CAV1^−/−^) breeders were obtained from Jackson Laboratory and were maintained in the Liu lab at Hunan TCM University. OTII mice (Jackson Laboratory, Bar Harbor, ME) were crossbred with CD45.1^+^ (Ly5.1) mice (Jackson Laboratory) to produce CD45.1 × OTII F1 mice in the Tang Lab at Taishan Medical University. All mice were maintained in specific pathogen-free (SPF) conditions on a standard diet and water and were used when they were approximately 6–8 weeks old.

### Design and Expression of Recombinant FlgE and FlgEM

The details for vector construction and expression of the WT PAO1 FlgE were previously reported in a Chinese journal^20^. In brief, the fragment encoding FlgE was amplified from genomic DNA extracted from the PAO1 strain (ATCC27853, American Type Culture Collection, Manassas, VA) using PCR. The primers were designed according to the genomic accession number AE004901 with addition of restriction enzyme sites *Nde*I and *Hind*III at the 5′ ends (forward: GGAATTCCATATGAGTTTCAACATCGGCCTG; reverse: TCCCAAGCTTGCGCAGGTTGATGATGGTCT) and synthesized by GenScript (Nanjing, China). PCR was performed with PCR MasterMix (Tiangen Biotech, Beijing, China) and the products were digested with restriction enzymes *Nde*I and *Hind*III, and the recovered fragments were cloned into the prokaryotic expression vector pET24a (Novagen, San Diego, CA) using the *Nde*I and *Hind*III sites. After routine transformation and plating, kanamycin-resistant clones were picked, and extracted plasmids were sequenced across both the target gene and the linkage areas, including the 6 × His-tag at the C-terminal end. The correct recombinant plasmid was named pET24a-*flgE*. To study the correlation between the structure and activity of FlgE, the two sites that were homologue with the two PhD-peptides from previous study^17^ (also refer to Fig. S1) were selected for mutation to generate FlgEM. The fragment coding for a similar reading frame as PAO1 FlgE was synthesized by Genewiz Biotechnology (Beijing, China), except that two mutations were introduced. The first mutation was a deleterious change to site B at nucleotides 1166383–1166403 of AE004901, namely the CCGCCGACCGTGACGCCGTTC string, and the second mutation was a substitution of site F (ACCCCGCCGACCTACGCCTGG, nucleotides 1166788–1133808 of AE004901) with GCAGCAGCA. These changes resulted in a seven-amino-acid deletion at site B (PPTVTPF, aa168–174) of NP_249771 and a substitution of seven amino acids at site F (TPPTYAW, aa303–309, NP_249771) with three consecutive alanines (AAA). The synthetic fragment was provided by the manufacturer in the cloning vector pUC57 between *Nde*I and *Hind*III sites. After digestion with restriction enzymes *Nde*I and *Hind*III, the fragment was recovered and cloned into pET24a as described above, the resulting plasmid being named pET24a*-flgEM*.

To obtain recombinant FlgE and FlgEM proteins, the pET24a*-flgE* and pET24a*-flgEM* plasmids were transformed into BL21(DE3) (Novagen) competent cells, and positive clones were grown in LB broth with 50 mg/L kanamycin under agitation at 225 rpm and 30 °C. When the OD_600_ was between 0.6–0.9, IPTG (Sigma, St Louis, MO) was added to 1 mM, and the culture was grown at 225 rpm and 16 °C for another 20 h. The culture was harvested using binding buffer (20 mM sodium phosphate, 0.5 M NaCl, 30 mM imidazole, pH 7.4) containing 0.03 g/L lysozyme and was ultrasonicated on ice. Pilot experiments demonstrated that the expressed proteins were soluble; therefore, the supernatant containing the overexpressed proteins was subjected to affinity chromatography using His-Trap Fast Flow Crude columns (GE Healthcare, Buckinghamshire, UK) in an Akata FPLC System (GE Healthcare) following the manufacturer’s instructions. The eluted proteins were run through a His-Trap Desalting Column (GE Healthcare), and then the Toxin Eraser^TM^ Endotoxin Removal Kit (GenScript, Piscataway, NJ) was used. The final elutions were confirmed to contain sole bands around the expected molecular weights (FlgE, MW 48336.08; FlgEM, MW 46992.53) respectively on a 12% SDS-PAGE gel. Totally three batches of FlgE or FlgEM were tested in this study and no significant variations were noted among these preparations (data not presented). Endotoxin in these protein preparations was measured using a chromogenic end-point tachypleus amebocyte lysate (CE-TAL) assay (Rongbo Biotechnology, Shanghai, China) and was present at less than 0.74 EU/mg, which was negligible for subsequent studies.

### Preparation of *P. aeruginosa* Flagellar Hooks

The flagellar hooks of WT PAO1 strain were purified following the procedure described by Limberger^47^. In brief, bacterial cultures were harvested in the late log phase of growth, resuspended in PBS, and sheared by forcing them through a 22-gauge hypodermic needle several times. Whole cells and debris were removed by low-speed centrifugation (8,000 × g, 15 min), and the cleared supernatant was subjected to ultracentrifugation at 75,000 × g for 1 h to obtain the purified filaments (with hook on). Then, the filaments were dissociated with an acidic buffer (50 mM glycine-Triton X-100, pH 2.5) at 25 °C for 1 h, and the hook was collected by ultracentrifugation (100,000 × g, 60 min). The pellet was resuspended in 10 mM Tris-HCl buffer, pH 7.5, and the protein concentration was measured using a kit from Applygen Technologies Inc. (Beijing, China) utilizing the bicinchoninic acid (BCA) methodology^48^.

### Effects of FlgE or FlgEM on Gene Expression in HCECs

For *in vitro* bioassay studies, HCECs (CRL-11135, ATCC) were seeded onto 24-well plates at 1.5 × 10^4^ in 500 μL Dulbecco’s Modified Eagle’s Medium (DMEM)/F12 supplemented with 10% heat-inactivated FBS (Invitrogen, Carlsbad, CA) at 37 °C. When 80–90% confluence was reached, the cells were starved for 4 h in culture medium without bovine serum, and then FlgE or FlgEM was added to 20 μg/mL, unless otherwise stated, for 4 h in triplicate. After incubation, cells were harvested with TRIzol (Qiagen, Valencia, CA), and RNA was extracted. RNA samples were subjected to microarray assay or real-time PCR (RT-qPCR) as described below.

### Lung Organoid Cultures

Lung organoid cultures were used as an *in situ* model for comparing the bioactivity levels of FlgE, FlgEM and flagellar hooks on isolated animal tissues. After sacrificing the mice, lungs were removed and perfused with cold PBS and were then instilled with pre-warmed 2% low-melting agarose in PBS to their approximate original size. After solidifying, the lungs were cut into 1-mm-thick slices using a customized utensil. The tissues were exposed to predetermined concentrations of recombinant FlgE, FlgEM, flagellar hooks, LPS, or vehicle (PBS) in 24-well plates containing serum-free DMEM. After routine culturing at 37 °C for 24 h, lung slices were collected and subjected to gene detection using RT-qPCR, as described above. In some cases, U0126 (Cat#9903, Cell Signal Technology, Danvers, MA) was added to a concentration of 10 µM during culture. For confirmation of CAV1 involvement in FlgE stimulation, CAV1^−/−^ mice were also used in parallel with WT mice. For all assays and in each treatment, duplicates were set. Three mice were used, and for each individual treatment, equal numbers of slices from each of the three mice were mixed and subjected to culture.

### Proinflammatory Activity of FlgE in Mice

To detect the *in vivo* proinflammatory effects of FlgE or FlgEM, C57BL/6 mice were treated intranasally with a 150-μL solution containing 120 μg of recombinant FlgE, FlgEM, LPS or vehicle PBS only. Six mice were included in each group. All animals were sacrificed 24 h later, and bronchoalveolar lavage was performed with three 1-mL aliquots of sterile saline solution. Cytokines in the lavage were measured using ELISA kits for murine IL1β, IL6 and CXCL1 (Cusabio Biotech, Wuhan, China), and serum MPO activity was measured using an MPO determination kit (Nanjing Jiancheng Corp., Nanjing, China). The lungs were removed and cut into halves. One half was fixed in 4% formaldehyde, embedded in paraffin and cut into 4-μm sections for hematoxylin and eosin (H&E) staining, while the other half was subjected to gene detection using RT-qPCR as described above.

### Adjuvanticity of FlgE on Soluble Antigen in Mice

To examine the potential adjuvanticity of FlgE in animals, OVA was used as a soluble antigen to immunize mice receiving OVA-specific T cells. In brief, spleens and inguinal lymph nodes were removed from CD45.1 × OTII F1 mice and were minced through a 70-μm nylon mesh to obtain single cells. The cells were labeled with eFluor450, CD16/32 and a mixture of antibodies (1 μg/mL each of CD45.1-FITC, CD4-Percp/Cy5.5, TCRvalpha2-APC/Cy7, TCRvbeta5-APC, CD62L-PE, and CD25-BV650, all from eBioscience, San Diego, CA) sequentially before being sorted with CD4^+^ MACS beads (Miltenyi Biotec GmbH, Bergisch Gladbach, Germany), and the recovered CD4^+^ cells were further sorted using a BD FACSAria II (BD Biosciences, San Jose, CA) for CD4^+^CD45.1^+^TCRvalpha2^+^TCRvbeta5^+^CD62L^+^CD25^−^ fractions. The purified naïve T cells were transferred to female C57BL/6 mice at 1 × 10^6^ cells/mouse via tail vein injection. Twenty-four hours later, the recipients were immunized via subcutaneous injection at the base of the tail with one of eight compositions in 100 μL, namely OVA (1 μg), OVA plus 50 μg CpG-1826 (both from Sigma, St Louis, MO), OVA plus 50 μg FlgE, OVA plus 100 μg FlgEM, 50 μg CpG-1826, 100 μg FlgE, 100 μg FlgEM, or PBS control. Three days later, the mice were sacrificed, and the draining inguinal lymph nodes were isolated, imaged and minced into a single-cell suspension for staining with CD45.1 and CD4 as described above. Cells were run on a BD FACSAria II and analyzed for eFluor450 intensity to reflect proliferation. To measure whether FlgE altered the humoral response of host animals to OVA, WT C57BL/6 mice were immunized with OVA (100 μg) alone, OVA plus 100 μg FlgE, OVA plus FlgEM 100 μg, OVA plus 50 μg CpG, or treated with 50 μg CpG, 100 μg FlgE, 100 μg FlgEM each alone as control, all via subcutaneous injection. Two weeks later, mice were sacrificed for serum harvest, and anti-OVA titers were measured using ELISA. For this purpose, 96-well plates were coated with 100 μL OVA in PBS (20 ng/mL) overnight at 4 °C and then blocked with 1% FBS in PBS buffer for 1 h at RT. Serial dilutions of sera were added and incubated for 2 h at RT, followed by washing and incubation with rat anti-mouse IgG1 or IgG2b-biotin antibody at 0.125 μg/mL for two hours. After washing, the wells were incubated with streptavidin-HRP for 30 min at RT. Then, the plates were washed three times, and a 3,3′,5,5′-tetramethylbenzidine (TMB) solution was added to each well. After 20 min of incubation, the reaction was stopped with 50 μl 1 M H_2_SO_4_, and the plates were read with a microplate reader at 450 nm.

### 3D Modeling of FlgE/FlgEM

The protein sequence of PAO1 FlgE (NP_249771 or Q9I4P9) was retrieved from the UniProt database (http://www.uniprot.org). The BLASTP program was subsequently used to search for FlgE homologues from the RCSB Protein Databank (http://www.pdb.org). Consequently, FlgE from *S. typhimurium* (PDB ID code: 3A69)^28^ was determined to have 35.7% identity and 54.5% similarity with the PAO1 FlgE, making it a tentative template for structural predictions of the latter. The 3D structure of FlgE was then modeled using the MODELER program in the Discovery Studio 4.0 package (Accelrys Inc., San Diego, CA, USA)^49^ and was optimized using energy minimization with the Amber force field and minimized gradually (hydrogens, side-chains, all) to alleviate any remaining bad steric contacts. The optimized 3D structure was finally evaluated using Procheck and Profiles-3D to examine the stereochemical quality and structural rationality.

### Microarray Assay

RNA extraction from HCECs and subsequent microarray procedures were performed by the Agilent-contracted Shanghai OE Biotech (Shanghai, China). In brief, total RNA was extracted using a QIAGEN RNeasy Mini Kit, and an Agilent 2100 Bioanalyzer was used to confirm that the RNA quality of all samples was sufficiently high, as reflected by RNA Integrity Numbers greater than 9.9 and 28S/18S ratios greater than 1.6. Two hundred nanograms of RNA was reverse transcribed into cDNA using a T7 promoter-tagged polyT primer. The resulting cDNA was transcribed into cRNA, during which the Cy3 and Cy5 fluorescent tags were incorporated into samples of FlgE-treated and medium control groups, respectively. Hybridization was performed on an Agilent SurePrint G3 Human Gene Expression 8 × 60 K array (Cat# G2543-60015, Agilent) using an Agilent G2545A Oven for 17 h at 65 °C. These arrays detected 34,745 protein-coding transcripts and 7660 LncRNA plus internal quality control probes. An Agilent G2505C Scanner was used to scan the hybridized arrays. Each array was scanned twice, at a resolution of 5 μm, photomultiplier tube (PMT), gain at 100% and 10%, and the scanned results were combined for final extraction using Feature Extraction. Extracted fluorescence data were normalized and analyzed using GENESPRING12.0 (Silicon Genetics, Redwood City, CA). Only the features marked as DETECTED in at least two of the three arrays were considered “expressed” and used in downstream analysis. For each expressed gene probe, an FlgE/Control ratio (i.e., fold change) was obtained for each array, and a P value was calculated for comparison of the fluorescence intensities in those two groups. An average fold change of greater than 1.50 or less than 0.667 for all three arrays with a P < 0.10 between FlgE and control groups was arbitrarily selected for judging significant upregulation or downregulation, respectively. Differentially expressed genes were annotated and clustered using DAVID (v6.7, NIH, Bethesda, MA), with the whole human genome as the background^50^. The microarray data are MIAME compliant and were deposited in the Gene Expression Omnibus (NBCI, NIH) with the accession number GSE58422.

### Real-time PCR (RT-qPCR)

RT-qPCR was used to measure the gene expression of specific genes at the mRNA level in either HCECs, organoids or lung samples. Total RNA was extracted using a NucleoSpin® RNA II Kit (MACHEREY-NAGEL, Düren, Germany) in our lab. Reverse transcription was performed using a PrimeScript™ First Strand cDNA Synthesis Kit (Takara, Otsu, Japan) with 900 ng total RNA, followed by TaqMan real-time PCR using an Applied Biosystems 7500 Real-Time PCR System (Applied Biosystems, Foster City). The sequences of primers and probes of target genes are listed in Supplementary Table S3, and B2M and ACTB were used as reference genes for human and mouse samples, respectively. Amplification was set to 10 s at 95 °C, followed by 40 two-step cycles (15 s at 95 °C and 1 min at 60 °C). After analysis with the included SDS System Software (Applied Biosystems), the Ct for each reaction was obtained. The average of three duplicates was used to calculate the relative Ct against B2M (ΔCt = Ct_gene_ − Ct_B2M/ACTB_) for each sample. Then, the average ΔCt from the three samples was used to calculate the ΔΔCt of the experimental samples against the PBS control (ΔΔCt = ΔCt_FlgE_ − ΔCt_PBS_). The relative expression of genes in the FlgE/FlgEM-treated samples was calculated as 2^−ΔΔCt^.

### Pull-down Assays and Candidate Proteins Confirmation

To identify potential receptors or conjugating molecules for FlgE, a homemade affinity chromatography column was utilized. In brief, 500 μg of purified recombinant FlgE was mixed with 100 μL His-agarose beads (recovered from a His-Trap FF Crude column) in binding buffer (20 mM Sodium Phosphate, 500 mM Sodium Chloride, 30 mM Imidazole, pH 7.4) and was incubated at 4 °C for 16 h. Unbound FlgE was removed by washing with PBS 3 times. Then, total membrane proteins were prepared from ~10^8^ HCECs using a Membrane Protein Extraction Kit (Bestbio, Shanghai, China) and were incubated with the FlgE-saturated agarose in 300 μL of PBS at 4 °C for 8 h with an agitation of approximately 20 rpm. The incubation of membrane proteins with balanced empty agarose beads was performed simultaneously as the negative control. Following five washes with PBS, the proteins bound to the agarose were eluted in 100 μL of 0.2 M glycine-HCl. After immediate neutralization with 15 μL of 1 M Tris-HCl, pH 9.1, the elution was concentrated into 20 μL using an ultrafiltration tube (Millipore, Bedford, MA) and then resolved on a 12% SDS-PAGE separation gel for 30 min. The gel was stained with G-250 dye. Six protein bands of the FlgE-membrane proteins incubation group with significant difference to the control were selected and cut, and then destained with 0.1% acetic acid, and subjected to in-gel digestion with trypsin. The resulting peptides were analyzed by nano-liquid chromatography-tandem mass spectrometry using a nanoACQUITY UPLC and SYNAPT G2 HD mass spectrometer (Waters Corporation, Milford, MA). Mass spectrometry data were acquired using the Data Dependent Analysis mode and processed with PLGS 2.4 software (Waters), and the resulting peak list was searched against the NCBInr Homo sapiens database using the MASCOT search engine (Matrix Science Inc., Boston, MA). This program sets 38 as the default cutoff for possible matches. In this study, however, proteins were considered potential FlgE-binding partners if at least one peptide had a score equal to or above an arbitrary score of 114 (i.e., three times the default cutoff score of 38).

To confirm the true presence of a specific protein suggested by the aforementioned mass spectrometry, here CAV1, Western blot was utilized on the pulled down proteins. At the same time, to evaluate the postulated specificity for FlgE-CAV1 interaction, FlgEM-conjugated agarose were prepared exactly like with FlgE-agarose described above. For comparison, same amount of HCEC membrane proteins were incubated with FlgE or FlgEM-saturated agarose to allow for binding at 4 °C for 8 h. The beads were washed with PBS, suspended in 1× SDS loading buffer and boiled for 10 min. The beads were spun down, and the supernatants were subjected to SDS-PAGE and transferred to a polyvinylidene difluoride membrane (Millipore, Billerica, MA). The membranes were blocked in 5% nonfat dry milk dissolved in Tris-buffered saline with Tween-20 (TBST; 20 mM Tris, pH 7.5, 0.5 mM NaCl, 0.05% Tween-20) for 1 h and were then incubated overnight with anti-CAV1 antibody (ab2910, Abcam, Cambridge Science Park, Cambridge, UK) and anti-His antibody (Transgen Biotech, Beijing, China) at 4 °C. After an agitating wash for 30 min, the membranes were incubated with HRP-conjugated Affinipure goat anti-rabbit IgG antibody or anti-mouse IgG antibody (Cell Signal Technology, CA) at room temperature for 1 h. After extensive washing, hybridized bands were detected with an Enhanced ChemiLuminescence Kit (Perkin-Elmer Life Sciences, Boston, MA) as instructed by the manufacturer. For loading control purposes, a mouse monoclonal anti-GAPDH antibody from Kangchen (Shanghai, China) and a rabbit anti-mouse IgG antibody from ZSGB-BIO Biosciences (Beijing, China) were utilized.

### Western Blot of FlgE-treated Lung Organoid Tissues

To explore the possible pathways affected by FlgE stimulation, protein samples were prepared from wild type and CAV1^−/−^ mice lung organoids treated with 5 μg/mL FlgE at different time points and subjected to routine Western blot with antibodies against phospho-Stat3, Stat3, phospho-Erk1/2, Erk1/2, phospho-p38, p38 (1:1000, Cell Signal Technology) and β-Tubulin (1:3000, Sanjian, Tianjin, China). Resulting bands were subjected to densitometry assay with ImageJ software (NIH, Bethesda, MD).

### Statistical Analysis

When applicable, the data are presented as the means ± SEM. For pair-wise comparisons, the non-parametric Mann-Whitney U test was used; for multi-group analysis, one-way ANOVA followed by Dunn’s procedure was performed. P-values < 0.05 were considered significant.

## Electronic supplementary material

Supplementary Information
