# Supplementary material for: Flagellar Hooks and Hook Protein FlgE Participate in Host Microbe Interactions at Immunological Level
Source: Sci Rep. 2017 May 3;7:1433. doi: 10.1038/s41598-017-01619-1 (PMC5431167; doi:10.1038/s41598-017-01619-1)
Supplement: Supplementary file 1 — Supplementary Information [file 41598_2017_1619_MOESM1_ESM.doc]

### Flagellar Hooks and Hook Protein FlgE Participate in Host Microbe Interactions at Immunological Level

Ying Shen, Lin Chen, Meixiang Wang, Dandan Lin, Zhongjie Liang, Peiqing Song, Qing Yuan, Hua Tang, Weihua Li, Kangmin Duan, Baiyan Liu, Ge Zhao, Yiqiang Wang


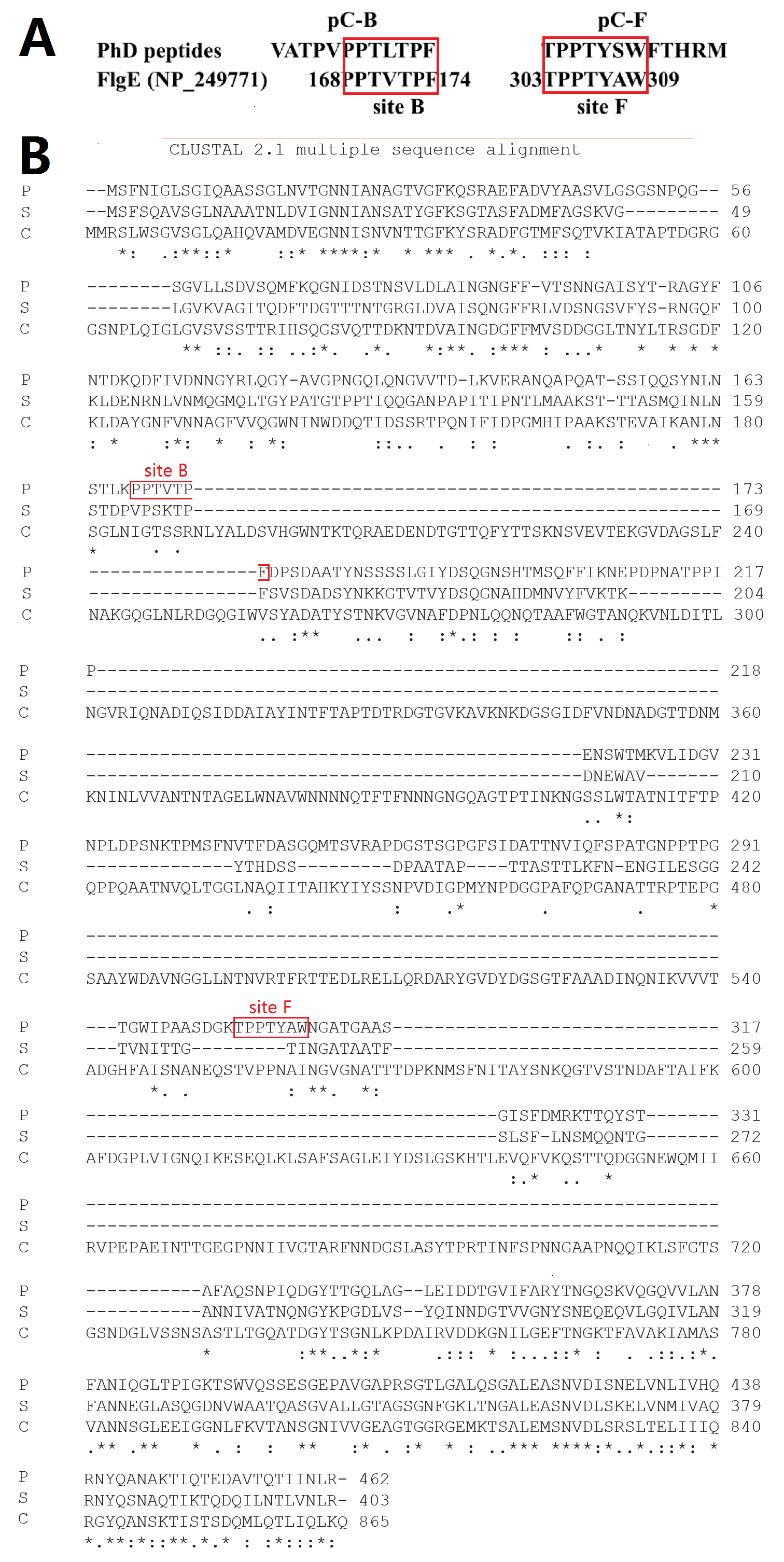
**Figure S1. Sequence alignments of FlgE from three common species.**

(A) PAO1 FlgE with the two starting peptides obtained with phage display screening. The upper line showed the sequences of the two 12-aa peptides obtained in an earlier phage-display screening study, while the lower line showed the accurate locations and sequences of sites B and F in PAO1 FlgE.

**(B)** CLUSTAL alignment of FlgE sequences of three common bacterial strains, namely PAO1 *(P)*, *S. typhi (S)* and *C. jejuni (C)*. Please note that neither site B nor F (in red boxes) is strictly conserved in FlgE sequences, indicating that the structure-activity correlation in each FlgE might be unique.

**(C)** CLUSTAL alignment of FliC filament flagellin sequences of PAO1 *(P)*, *S. typhi (S)* and *C. jejuni (C)*. Please note that one of the proposed TLR5 recognition site (in red box) for *S typhi* flagellin is not strictly conserved in all flagellin sequences, indicating that the structure-activity correlation in each flagellin-TLR5 complex might be unique as well.

**C (FIGURE S1, continued)**


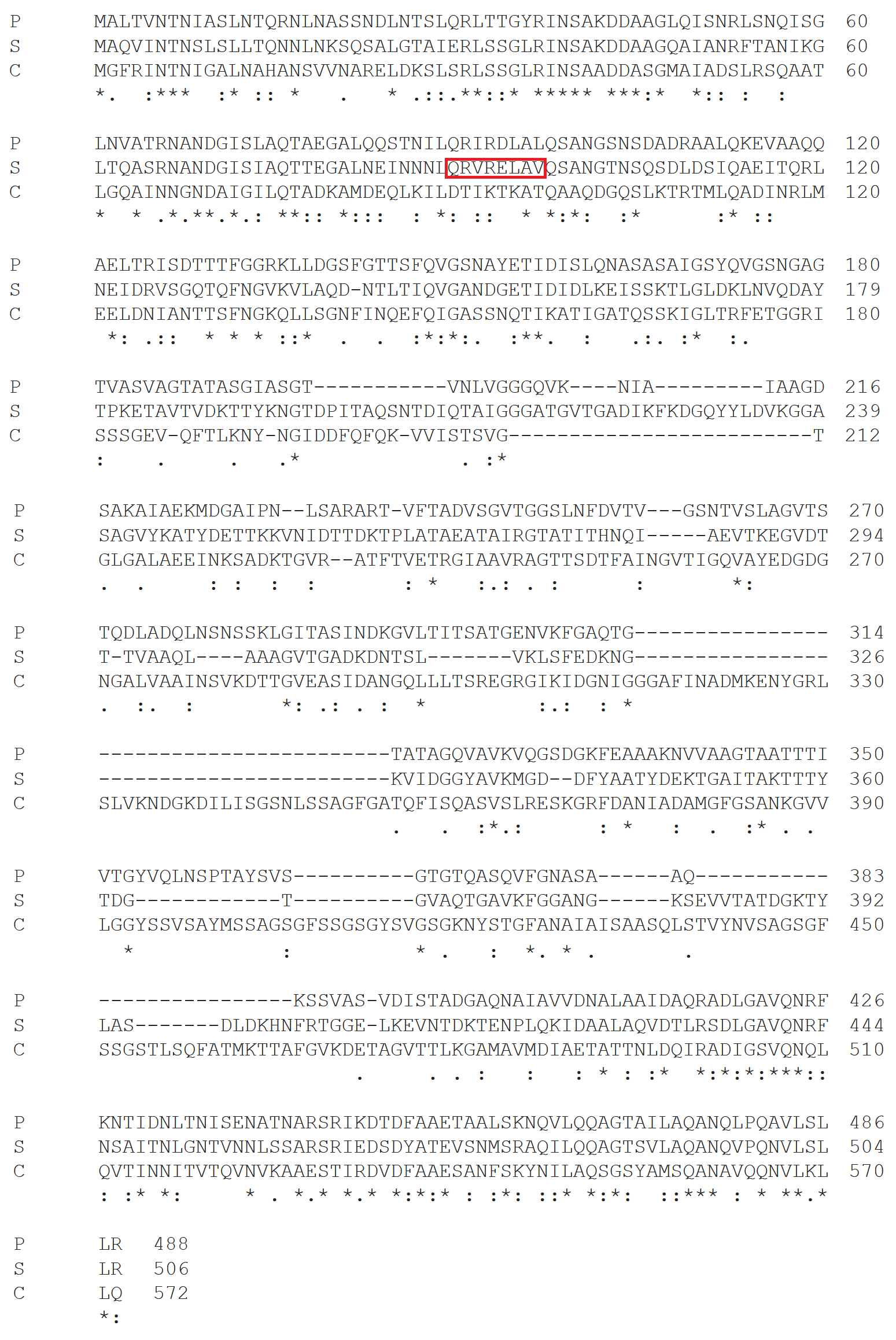


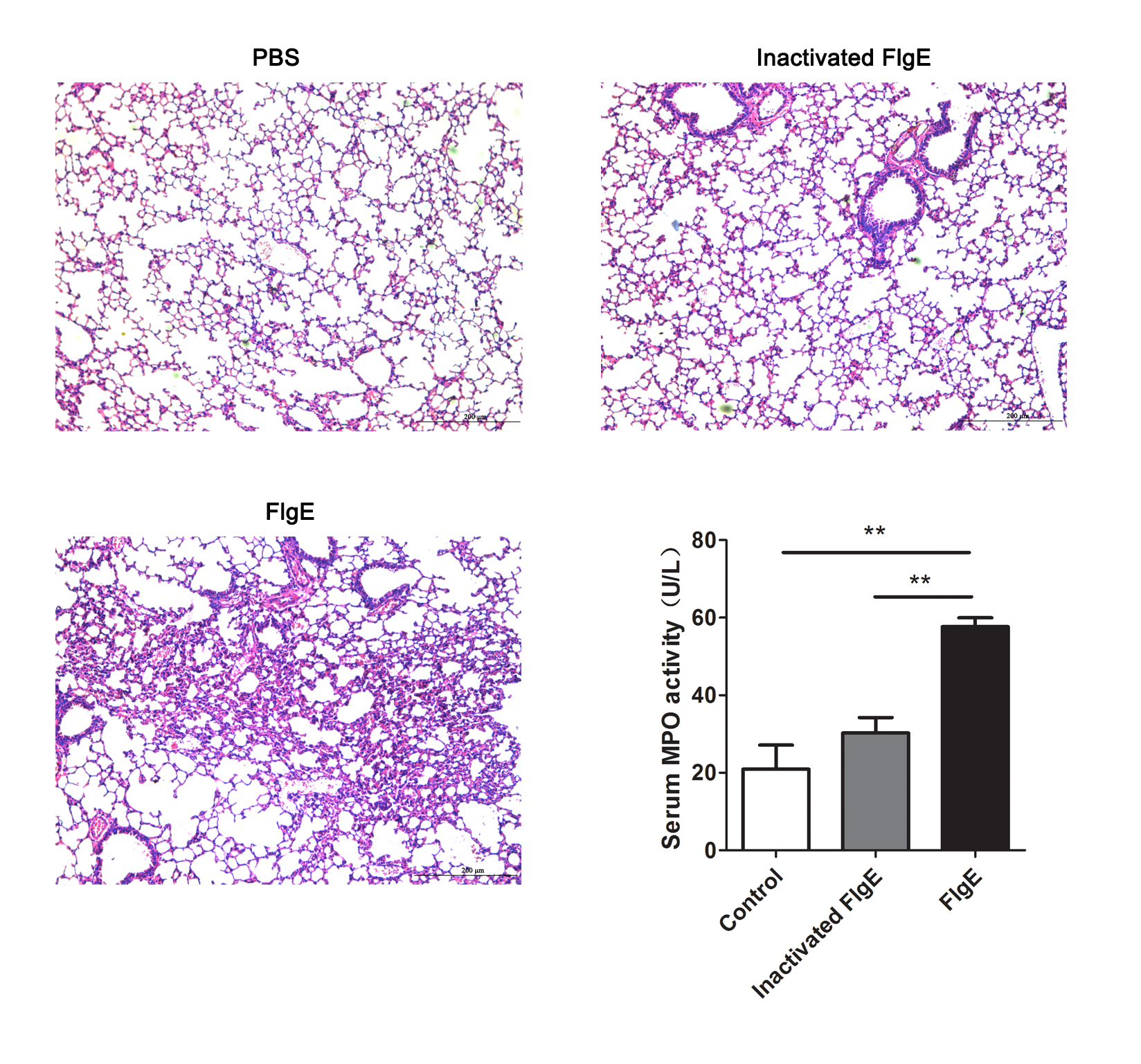


**Figure S2. Denaturation of recombinant FlgE abolished its proinflammatory activity in mice.** C57Bl/6 mice were all intranasally administered 120 μg native or denatured recombinant FlgE in a 150-μL volume. Twenty-four hours later, the animals were sacrificed by bleeding under anesthesia. Myeloperoxidase in serum was measured and, lungs were subjected to fixation and routine histological observation.


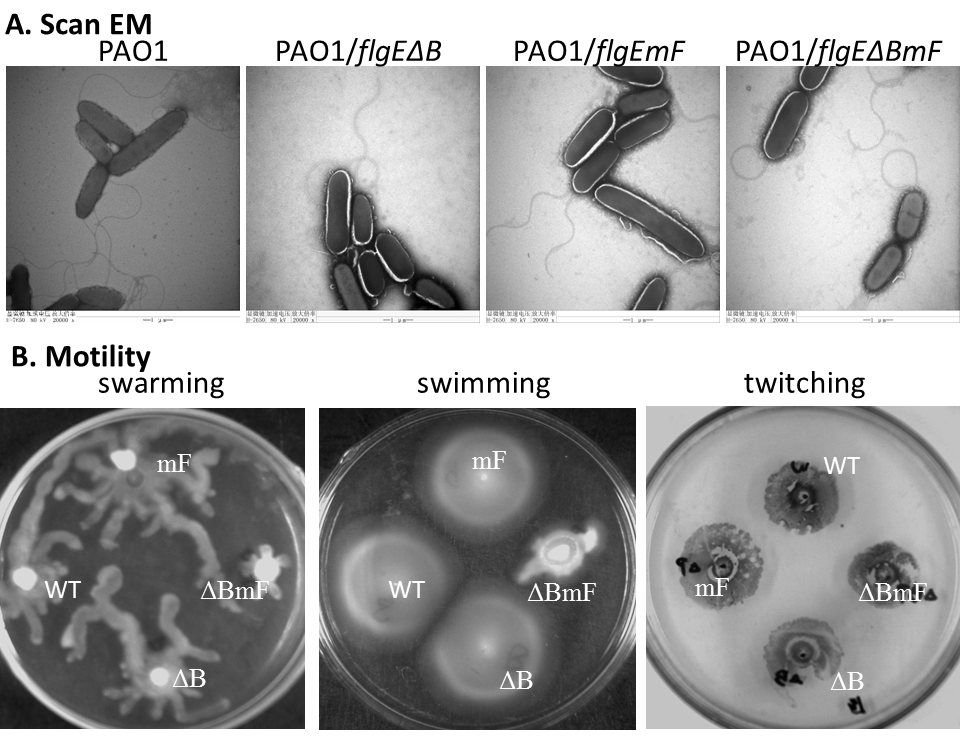
**Figure S3. Requirement for sites B and F for flagellar motility.** PAO1 strains with alteration of site B and/or site F were constructed using the *sacB*-based strategy. Gross appearance (A) and motility (B) of bacteria were examined using transmission electron microscopy and agarose inoculation respectively. Main results: though a portion of PAO1/*flgEΔBmF* strain retained apparently normal flagella, their swimming ability was approximately totally lost.

***Material and Methods for Figure S3***

***Construction of PAO1 Mutants***

The *sacB*-based strategy (Schweizer and Hoang, 1995) was employed to construct PAO1 mutant strains with mutations in the *flgE* gene. All vectors and host bacteria were maintained in Duan Lab at the University of Manitoba, Canada (Shen et al., 2012). The fragment encoding *flgEM* was recovered from pET24a*-flgEM* (refer to main text for detail of pET24a- *flgEM*) with *Sph*I and *Hind*III and ligated into the pEX18Amp vector to obtain the suicide plasmid pEX18Amp-*ΔflgE.* After transformation into *E. coli* DH10B cells, the resulting transformant (DH10B/pEX18Amp-*ΔflgE*) was used as a donor for triparental mating with *E. coli* pRK2013 as the helper strain and WT PAO1 as the recipient strain. After routine culture and expansion, PCR with different primers was utilized to identify the expected recombinant, and a mutant with a site F mutation, namely PAO1/*flgEmF,* was obtained. In PAO1/*flgEmF,* the 7 amino acids at site F of FlgE were substituted with AAA. Then, using DH10B/pEX18Amp-*ΔflgE* as the donor and PAO1/*flgEmF* as the recipient, another triparental mating was performed, and PAO1/*flgEΔBmF* was obtained, where site B was deleted in addition to the site F mutation, exactly as was created in FlgEM. For single site B deletion mutant, the upstream and downstream fragments were amplified by PCR and fused with pEX18Amp by EsayGeno VI-201 seamless cloning kit [TIANGEN BIOTECH (BEIJING) CO., LTD]. Triparental mating was performed as above. All PAO1 mutants were subjected to sequencing to confirm the presence and accuracy of expected mutations in the *flgE* gene. The WT and mutant PAO1 strains were subjected to electron microscopy and motility assays as below.

***Electron Microscopy***

Log-phase bacteria were pelleted and resuspended in 2% glutaraldehyde in PBS. A drop of this suspension was placed onto carbon-coated copper grids, which were then air-dried and negatively stained with 2% phosphotungstic acid. The samples were examined with a Hitachi H-7650 Transmission Electron Microscope (Hitachi High-Technologies Corporation, Tokyo, Japan).

***Motility Assay***

This was done according to a previous protocol (Rashid and Kornberg, 2000) with modifications. For ease of comparison, the four strains were inoculated side-by-side in each plate. Briefly, bacterial cells grown in LB medium overnight at 37°C were adjusted to an OD_600_ of 0.5, and 2 μL was used for each inoculation in all assays. For the swimming assay, plates made of 0.3% agarose in tryptone broth (10 g/L tryptone, 5 g/L NaCl) were inoculated and cultured at 37°C overnight. For the swarming assay, 0.5% agarose in nutrient-glucose broth (8 g/L nutrient broth, 5 g/L glucose) was used, and the plates were air-dried overnight before use. Then, 2-μL cultures were inoculated onto the plates and were cultured for 24 h. For the twitching assays, the cells were inoculated underneath plates cast with 1% agarose in regular LB broth (10 g/L tryptone, 5 g/L yeast extract, 10 g/L NaCl). After overnight incubation at 37°C, the agar was carefully removed, and the plate was stained with Coomassie blue to stain the motility zone. At the ends of all assays, pictures were taken for direct comparisons of the strains, but no quantitative calculations were attempted.

**Table S1. Pathways Enriched in Differentially Expressed Genes in FlgE-Stimulated HCECs**

| Term | Count | % | P Value* | Genes | Fold Enrichment |
| --- | --- | --- | --- | --- | --- |
| **Up-Regulated Genes** | | | | | |
| hsa04060:Cytokine-cytokine receptor interaction | 17 | 6.97 | 2.14E-06 | *CXCL1, CSF2, TNFRSF21, CCL3, IL6, CXCL2, TNFSF14, IL6R, IL24, CCL4, IL11, INHBA, TNFRSF11B, IL23A, IFNE, IL1B, IFNGR2* | 4.07 |
| hsa04630:Jak-STAT signaling pathway | 13 | 5.33 | 4.22E-06 | *CSF2, IL6, SOCS2, SOCS3, IL6R, IL24, CISH, IL11, IL23A, IFNE, SPRED2, MYC, IFNGR2* | 5.27 |
| hsa04621:NOD-like receptor signaling pathway | 7 | 2.87 | 3.83E-04 | *CXCL1, IL6, CXCL2, NFKBIA, IL1B, TNFAIP3, BIRC3* | 7.09 |
| hsa04610:Complement and coagulation cascades | 7 | 2.87 | 6.84E-04 | *PLAT, THBD, C3, CFB, C5, BDKRB1, BDKRB2* | 6.37 |
| hsa04010:MAPK signaling pathway | 13 | 5.33 | 8.39E-04 | *RELB, ELK1, FOS, DUSP4, BDNF, DUSP1, JUN, DUSP16, MAP3K8, IL1B, MYC, MAP3K12, DUSP6* | 3.06 |
| hsa04620:Toll-like receptor signaling pathway | 8 | 3.28 | 9.59E-04 | *FOS, CCL3, IL6, JUN, MAP3K8, NFKBIA, IL1B, CCL4* | 4.97 |
| **Down-Regulated Genes**** | | | | | |

* EASE score threshold was set at 0.01.

** In this setting, not any pathways were enriched for down-regulated genes.

**Table S2. Genes with greater than 2-fold changes in gene expression**

| GenBank Accession | Gene Symbol | Gene Description | Fold Change* |
| --- | --- | --- | --- |
| NM_001511 | *CXCL1* | chemokine (C-X-C motif) ligand 1 | 54.66±6.79  10.71±0.73 |
| NM_002852 | *PTX3* | pentraxin 3, long | 22.93±1.35 |
| NM_000576 | *IL1B* | interleukin 1, beta | 8.78±0.97 |
| NM_002089 | *CXCL2* | chemokine (C-X-C motif) ligand 2 | 8.76±1.12  5.53±0.60 |
| NM_000600 | *IL6* | interleukin 6 | 8.28±1.82 |
| NM_006290 | *TNFAIP3* | tumor necrosis factor, alpha-induced protein 3 | 7.78±0.11 |
| NM_002964 | *S100A8* | S100 calcium-binding protein A8 | 5.64±0.23 |
| NM_007329 | *DMBT1* | deleted in malignant brain tumors 1, transcript variant 2 | 4.63±0.65 |
| NM_001710 | *CFB* | complement factor B | 4.49±0.41 |
| NM_000623 | *BDKRB2* | bradykinin receptor B2 | 4.04±0.26 |
| NM_000710 | *BDKRB1* | bradykinin receptor B1 | 3.97±0.20 |
| NM_001136485 | *C11orf86* | chromosome 11 open reading frame 86 | 3.96±1.37 |
| NM_001570 | *IRAK2* | interleukin-1 receptor-associated kinase 2 | 3.77±0.18 |
| NM_005555 | *KRT6B* | keratin 6B | 3.56±0.85 |
| NM_002543 | *OLR1* | oxidized low density lipoprotein (lectin-like) receptor 1 | 3.22±0.25 |
| NM_001165 | *BIRC3* | baculoviral IAP repeat containing 3 | 3.20±0.21 |
| NM_138433 | *KLHDC7B* | kelch domain containing 7B | 3.19±0.09  2.77±0.59 |
| NR_002715 | *RN7SL1* | RNA, 7SL, cytoplasmic 1 | 3.08±2.4 |
| NM_004666 | *VNN1* | vanin 1 | 3.05±0.14 |
| NM_001185156 | *IL24* | interleukin 24 | 3.01±0.15 |
| NM_019060 | *CRCT1* | cysteine-rich C-terminal 1 | 3.00±0.47 |
| NM_021732 | *AVPI1* | arginine vasopressin-induced 1 | 2.97±0.15  2.40±0.19 |
| NM_015714 | *G0S2* | G0/G1switch 2 | 2.97±0.16 |
| NM_000758 | *CSF2* | colony-stimulating factor 2 | 2.88±0.36 |
| NM_000214 | *JAG1* | jagged 1 | 2.88±0.15 |
| NM_002747 | *MAPK4* | mitogen-activated protein kinase 4 | 2.81±0.28 |
| NM_001946 | *DUSP6* | dual specificity phosphatase 6 | 2.78±0.19 |
| NR_024390 | *LOC646999* | akirin 1 pseudogene | 2.77±0.42 |
| NM_014409 | *TAF5L* | TAF5-like RNA polymerase II, p300/CBP-associated factor (PCAF)-associated factor, 65 kDa | 2.73±1.80 |
| NM_000361 | *THBD* | thrombomodulin | 2.73±0.40 |
| NM_152997 | *C4orf7* | chromosome 4 open reading frame 7 | 2.66±0.50 |
| NM_006732 | *FOSB* | FBJ murine osteosarcoma viral oncogene homolog B, transcript variant 1 | 2.60±0.47 |
| NM_019593 | *GPCPD1* | glycerophosphocholine phosphodiesterase GDE1 homolog (*S. cerevisiae*) | 2.57±0.14 |
| NM_032413 | *C15orf48* | chromosome 15 open reading frame 48, transcript variant 2 | 2.56±0.15 |
| NM_030754 | *SAA2* | serum amyloid A2, transcript variant 1 | 2.54±0.06 |
| NM_016584 | *IL23A* | interleukin 23, alpha subunit p19 | 2.47±0.17 |
| NM_006509 | *RELB* | v-rel reticuloendotheliosis viral oncogene homolog B | 2.43±0.05 |
| NM_005261 | *GEM* | GTP-binding protein overexpressed in skeletal muscle, transcript variant 1 | 2.42±0.17 |
| NM_004073 | *PLK3* | polo-like kinase 3 | 2.40±0.45 |
| NM_005461 | *MAFB* | v-maf musculoaponeuroticfibrosarcoma oncogene homolog B (avian) | 2.37±0.32  2.11±0.27 |
| NM_017510 | *TMED9* | transmembrane emp24 protein transport domain containing 9 | 2.35±1.50 |
| NM_001040619 | *ATF3* | activating transcription factor 3, transcript variant 4 | 2.32±0.65 |
| NM_003679 | *KMO* | kynurenine 3-monooxygenase | 2.31±0.47 |
| NM_001657 | *AREG* | amphiregulin (AREG), mRNA [NM_001657] | 2.26±0.49  2.04±0.32 |
| NM_133639 | *RHOV* | ras homolog gene family, member V | 2.22±0.11 |
| NM_018689 | *KIAA1199* | KIAA1199 | 2.22±0.25 |
| NM_020529 | *NFKBIA* | nuclear factor of kappa light polypeptide gene enhancer in B-cells inhibitor, alpha | 2.19±0.12 |
| NM_001166692 | *C11orf91* | chromosome 11 open reading frame 91 | 2.19±0.46 |
| AF289590 | *LOC100130744* | clone pp7583 unknown mRNA | 2.16±0.99 |
| NM_002983 | *CCL3* | chemokine (C-C motif) ligand 3 | 2.15±0.74 |
| NM_024017 | *HOXB9* | homeobox B9 | 2.13±0.76 |
| NM_002467 | *MYC* | v-myc myelocytomatosis viral oncogene homolog (avian) | 2.09±0.15 |
| NM_020801 | *ARRDC3* | arrestin domain containing 3 | 2.07±0.43 |
| NM_003463 | *PTP4A1* | protein tyrosine phosphatase type IVA, member 1 | 2.06±0.12 |
| NM_002928 | *RGS16* | regulator of G-protein signaling 16 | 2.05±0.21 |
| AK310078 | *SAT1* | spermidine/spermine N1-acetyltransferase 1 | 2.04±0.31 |
| NM_021101 | *CLDN1* | claudin 1 | 2.04±0.37 |
| NM_005252 | *FOS* | FBJ murine osteosarcoma viral oncogene homolog | 2.03±0.15 |
| NM_005229 | *ELK1* | ELK1, member of ETS oncogene family, transcript variant 2 | 2.03±0.61 |
| NM_003713 | *PPAP2B* | phosphatidic acid phosphatase type 2B | 2.03±0.36 |
| NM_003897 | *IER3* | immediate early response 3 | 2.01±0.19 |
| NM_003442 | *ZNF143* | zinc finger protein 143 | 0.50±0.07 |
| NM_024511 | *HAUS3* | HAUS augmin-like complex, subunit 3 | 0.50±0.16 |
| NM_003810 | *TNFSF10* | tumor necrosis factor (ligand) superfamily, member 10, transcript variant 1 | 0.49±0.08 |
| NM_173649 | *C2orf61* | chromosome 2 open reading frame 61, transcript variant 2 | 0.49±0.08 |
| XM_003119451 | *LOC100507699* | PREDICTED: vesicle-fusing ATPase-like | 0.49±0.19 |
| NM_198699 | *KRTAP10-12* | keratin-associated protein 10-12 | 0.48±0.09 |
| NM_030641 | *APOL6* | apolipoprotein L | 0.48±0.40 |
| X58736 |  | human mRNA for T cell receptor V alpha gene segment V-alpha-w23, clone IGRa01 | 0.47±0.04 |
| AF498274 | *LRRC7* | leucine-rich repeat containing 7 | 0.46±0.02 |
| DW407923 | *SCARNA23* | HHAGE000653 human liver regeneration after partial hepatectomy cDNA | 0.42±0.24 |
| NM_052892 | *PKD1L2* | polycystic kidney disease 1-like 2, transcript variant 1 | 0.41±0.10  0.38±0.10 |
| NM_014722 | *FAM65B* | family with sequence similarity 65, member B, transcript variant 1 | 0.38±0.04 |
| AK057217 |  | cDNA FLJ32655 fis, clone TESTI1000025, weakly similar to *M. musculus* testis-specific protein, DDC8 | 0.34±0.15 |

* For those genes with two or more probes in the array setting, all applicable data were included.

**Table S3. Sequences of primers and probes used for real time-PCR**

| Gene (ID) | Primers and probes |
| --- | --- |
| *hB2M*  (NM_004048) | F, 5´-TAGCTGTGCTCGCGCTACTCT-3´  R, 5´-TTCTCTGCTGGATGACGTGAGTAA-3´  Probe, 5´-CTGGAGGCTATCCAGCGTACTCCA-3´ |
| *hIL8*  (NM_000584) | F, 5´-GGCAGCCTTCCTGATTTCTG-3´  R, 5´-TGCACTGACATCTAAGTTCTTTAGCA-3´  Probe, 5 ´-TGTGTGAAGGTGCAGTTTTGCCAAGG-3 ´ |
| *hIL6*  (NM_000600) | F, 5´-CCCCCAGGAGAAGATTCCAA-3´  R, 5´-TCAATTCGTTCTGAAGAGGTGAGT-3´  Probe, 5´-ATGTAGCCGCCCCACACAGACAG-3 ´ |
| *hCXCL1*  *(NM_001511)* | F, 5´-TATTTCTGAGGAGCCTGCAA-3´  R, 5´-ATCTCATTGGCCATTTGCTT-3´  Probe, 5´-CGCCAGCCTCTATCACAGTGGC-3´ |
| *hIL1β*  *(NM_000576)* | F, 5´- ACAGATGAAGTGCTCCTTCCA -3´  R, 5´- GTCGGAGATTCGTAGCTGGAT -3´  Probe, 5´- CTCTGCCCTCTGGATGGCGG -3´ |
| *hCXCL2*  *(NM_002089)* | F, 5´-CATCGCCCATGGTTAAGAA-3´  R, 5´-TCAGGAACAGCCACCAATAA-3´  Probe, 5´-TGGCAAATCCAACTGACCAGAAGG-3´ |
| *hIL23*  *(NM_016584)* | F, 5´-AACTGAGGGAACCAAACCAG-3´  R, 5´-ATCTCTGCCCACTTCCACTT-3´  Probe, 5´-CCTGATTCTCTCTGTTCAGCGCGT-3´ |
| *hIL24*  *(NM_001185156)* | F, 5´-AAGCAGATCCTCAATAAACATTTC-3´  R, 5´-ACCAAGGGAAAGGGATGATG-3´  Probe, 5´-TCCCACCCACACTCGCCAGC-3´ |
| *mIL1β*  *(NM_008361)* | F, 5’-CAACCAACAAGTGATATTCTCCATG-3’  R, 5’-GATCCACACTCTCCAGCTGCA-3’  Probe, 5’-CTGTGTAATGAAAGACGGCACACCCACC-3’ |
| *mIL6*  *(NM_031168)* | F, 5’-TCGGAGGCTTAATTACACATGTTC-3’  R, 5’-CAAGTGCATCATCGTTGTTCATAC-3’  Probe, 5’-CAGAATTGCCATTGCACAACTCTTTTCTCA-3’ |
| *mCXCL1*  *(NM_008176)* | F, 5’-CCGAAGTCATAGCCACACTC-3’  R, 5’-TTTTCTGAACCAAGGGAGCTT-3’  Probe, 5’-AAGGCAAGCCTCGCGACCAT-3’ |
| *mACTB*  *(NM_007393)* | F, 5’-GCAAGCAGGAGTACGATGAG-3’  R, 5’-CCATGCCAATGTTGTCTCTT-3’  Probe, 5’-TCCATCGTGCACCGCAAGTG-3’ |

**REFERENCES TO SUPPLEMENTARY MATERIALS**

Rashid, M. H., and Kornberg, A. (2000). Inorganic polyphosphate is needed for swimming, swarming, and twitching motilities of Pseudomonas aeruginosa. Proc Natl Acad Sci U S A *97*, 4885-4890.

Schweizer, H. P., and Hoang, T. T. (1995). An improved system for gene replacement and xylE fusion analysis in Pseudomonas aeruginosa. Gene *158*, 15-22.

Shen, L., Gao, X., Wei, J., Chen, L., Zhao, X., Li, B., and Duan, K. (2012). PA2800 plays an important role in both antibiotic susceptibility and virulence in Pseudomonas aeruginosa. Curr Microbiol *65*, 601-609.
